# Microstructure and Mechanical Properties of Inconel 740H after Long-Term Service

**DOI:** 10.3390/ma11112130

**Published:** 2018-10-30

**Authors:** Adam Zieliński, Marek Sroka, Tomasz Dudziak

**Affiliations:** 1 Institute for Ferrous Metallurgy, K. Miarki 12-14, 44-100 Gliwice, Poland; azielinski@imz.pl; 2 Institute of Engineering Materials and Biomaterials, Silesian University of Technology, Konarskiego 18a, 44-100 Gliwice, Poland; 3 Foundry Research Institute, Zakopiańska 73, 30-418 Krakow, Poland; tomasz.dudziak@iod.krakow.pl

**Keywords:** Inconel 740H, microstructure, precipitates, mechanical properties, superalloys

## Abstract

Inconel 740H is a nickel-based alloy for pressure components of ultra-supercritical boilers. Its chemical composition and strengthened matrix, as well as corrosion resistance, provide the highest creep resistance among the materials recommended for use in high-performance pressure components of power units. This paper investigates the changes in the microstructure and mechanical properties after ageing at 700 and 750 °C for 1000, 10,000, 20,000, and 30,000 h. Observation of the microstructure was performed using scanning and transmission electron microscopy. The identification of existing precipitates was conducted by X-ray phase analysis. The effects of time and ageing at elevated temperatures on the mechanical properties and precipitation process in the test alloy are discussed. The presented results are part of the material characteristics of the new-generation alloys to be used in the design of pressure equipment for steam boilers, as well as in diagnostic work during operation.

## 1. Introduction

Currently, power production throughout the world is approximately 40% coal combustion based. Since the end of the 20th century, great efforts have been made to modernize energy production as well as improving less efficient power generators now in circulation [1]. Reasons for such necessities are correlated with environmental protection and the development of alternative energy sources. Unfortunately, these alternatives have yet to become the primary energy source around the world. Hence, research of modern steels and alloys for high-performance energy engineering cannot be neglected [2,3,4].

Inconel 740H is a derivative of Nimocnic 263 nickel superalloy. After the modernization of the alloy’s chemical composition, by the refinement of the concentration relationship between Nb, Ti, and Al and the reduction of B and Si concentration, Inconel 740H was produced. It contains ca. 20% Cr, 20% cobalt, 2% niobium, and 1.8% titanium [2]. Due to its high chromium content, Inconel 740H possesses excellent high-temperature corrosion resistance and high strength properties. This is the result of solid-solution strengthening with Co and Mo, as well as M_23_C_6_ carbides and intermetallic phase γ, which are precipitated during the operation under creep conditions at 700–800 °C [2,5,6,7,8].

Inconel 740H is mostly used for loaded thin-walled elements of pater superheaters and thick-walled chambers and pipelines of ultra-critical steam boilers (700–760 °C, ca. 35 MPa). It shows high creep strength compared to other nickel-based alloys (i.e., 210 and 120 MPa at 700 and 750 °C, respectively, for 100,000 h) [2].

The materials used in the construction of steam boilers are required to show not only the adequate creep strength and corrosion resistance in steam and exhaust gas atmosphere, but also high mechanical properties both at room and elevated temperatures [9,10,11]. The process of changes in the performance of these materials, and in their microstructure, is described by the materials characteristics. These characteristics, in combination with other methods for assessing their exhaustion degree, are utilized in generating a safe product, operating both within and beyond the design service time [12,13,14].

Furthermore, recent studies on the Inconel 740H alloy have shown it has good resistance to oxidation in steam at 750 °C, due to the formation of a compact layer of Cr_2_O_3_ protective oxide on its surface. It also depends on internal oxidation changes occurring, which affects the adhesion of the oxide layer to the substrate and resistance to thermal shock during operation [7,8,15].

Changes in the microstructure, especially those occurring in the surface layer, may have a strong effect on the strength properties of Inconel 740H. These favor the formation of hard internal oxide particles and the reduction in the extent of the γ’ phase at the surface. Possible internal stresses due to the growth of oxide inclusions may also contribute to a decrease in creep strength and crack resistance.

Our study complements numerous publications on the corrosion resistance of Inconel 740H, in which the effect of the long-term impact of elevated temperature on performance stability of the test alloy were not examined [7,8,16,17,18].

## 2. Materials and Methods 

The material used in the investigations was Inconel 740H (NiCr_25_Co_20_TiAlNb), delivered in the form of tube samples of ϕ 31.8 × 6.3 mm. The chemical composition of the investigated alloy, with reference to the requirements of the standard, is presented in Table 1.

The microstructure of Inconel 740H was examined using the Inspect F light and scanning electron microscope (SEM) (FEI, Hillsboro, OR, USA) on conventionally-prepared electrolytically-etched metallographic microsections, and with the TITAN 80-300 transmission electron microscope (TEM) (FEI, Hillsboro, OR, USA) using thin foils. Analysis of the precipitation processes was conducted using X-ray analysis of carbide isolates and with thin foils using selective electron diffraction.

The investigations of the mechanical properties in the static tensile test at room temperature were conducted using the tensile testing machine (Zwick, Ulm, Germany) with 200 kN max load. Hardness measurements were performed using Vickers method, with a hardness testing machine that had a 10 kg indenter load.

Quantitative analysis of the precipitates was conducted with an image analysis system. The image analysis system was calibrated using the scale marker in photos. Calibration coefficient: One pixel = 0.040 µm. The above-mentioned investigations were performed on the material in the as-received condition and after long-term ageing. Aging was carried out in furnaces (Institute for Ferrous Metallurgy, Gliwice, Poland) under an air atmosphere at 700 and 750 °C, with an accuracy of 0.5 °C and a duration of 1000, 10,000, 20,000, and 30,000 h.

## 3. Results

### 3.1. Microstructure

**Figure 1:**
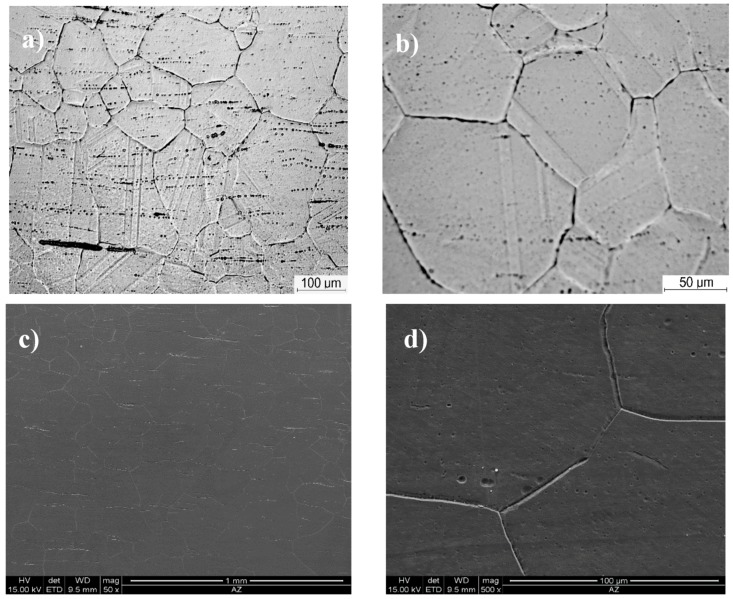
Microstructure of Inconel 740H in the as-received condition: (**a**,**b**) Light microscope (LM); (**c**,**d**) scanning electron microscope (SEM).

The microstructure of Inconel 740H in the as-received condition (solution annealing heat treatment at 1150 °C/0.5 h) observed with the light and scanning electron microscope is shown in Figure 1. The test material was characterized by the austenitic matrix with numerous annealing twins and single primary precipitates of varying size arranged in bands. The measured average grain diameter was approximately 100 µm (similar to that in the ASTM cards).

**Figure 2:**
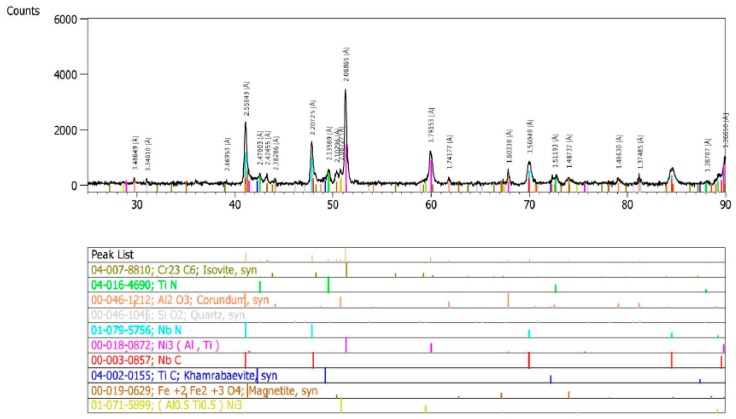
X-ray diffractogram of precipitate isolate in Inconel 740H in the as-received condition.

The qualitative X-ray phase analysis of the precipitates in the test alloy, in the as-received condition, showed the occurrence of niobium and titanium carbides and nitrides [M(C,N)], Cr_23_C_6_ carbides, and γ’ phase (Figure 2) [19,20].

**Figure 3:**
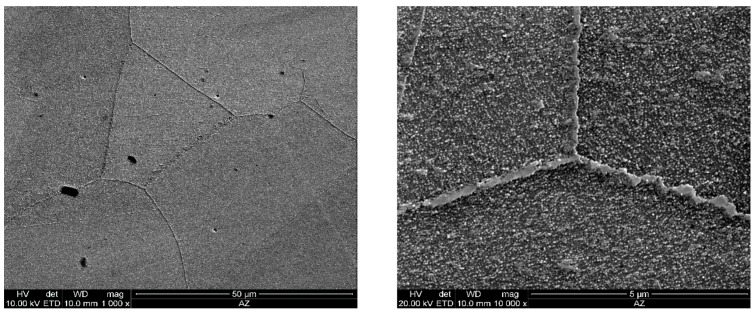
Scanning electron microscope analysis of the microstructure of Inconel 740H after ageing at 700 °C for 1000 h.

**Figure 4:**
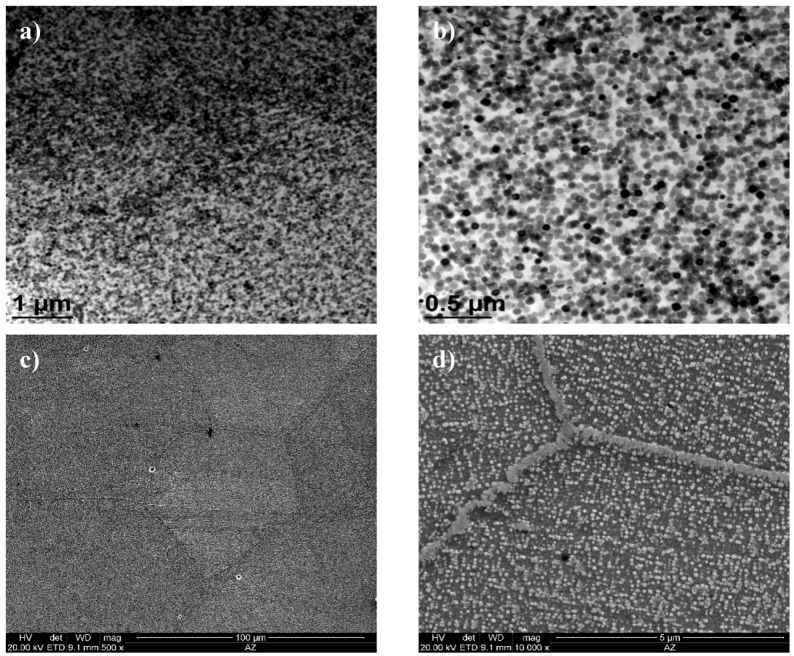
Microstructure of Inconel 740H after ageing at 750 °C for 1000 h, observed: (**a**,**b**) In transmission electron microscope; (**c**,**d**) in scanning electron microscope.

**Figure 5:**
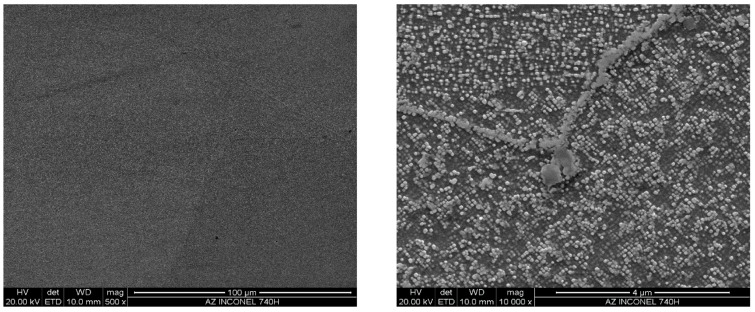
Scanning electron microscope analysis of the microstructure of Inconel 740H after ageing at 700 °C for 10,000 h.

**Figure 6:**
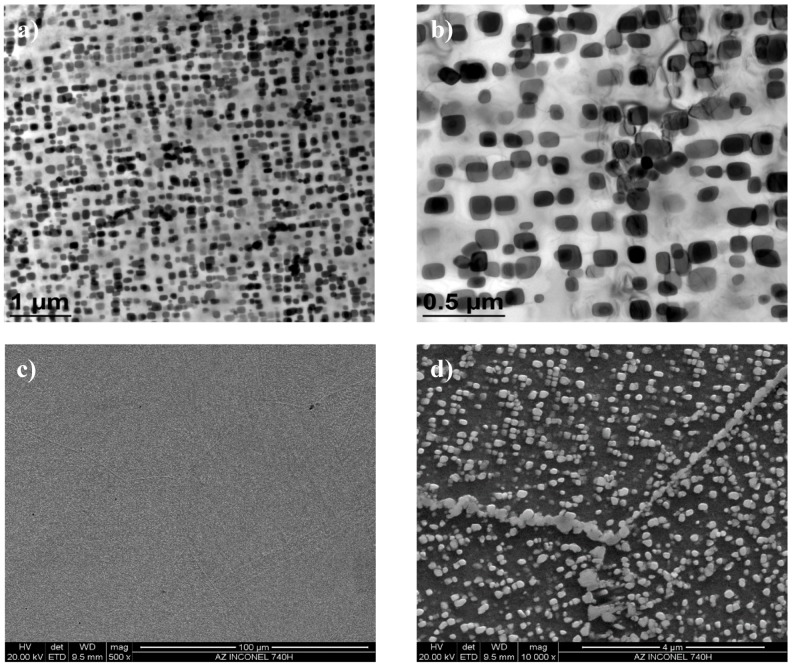
Microstructure of Inconel 740H after ageing at 750 °C for 10,000 h, observed: (**a**,**b**) In transmission electron microscope; (**c**,**d**) in scanning electron microscope.

**Figure 7:**
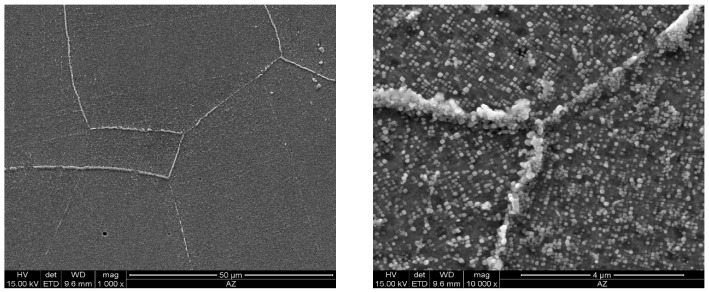
Scanning electron microscope analysis of the microstructure of Inconel 740H after ageing at 700 °C for 20,000 h.

**Figure 8:**
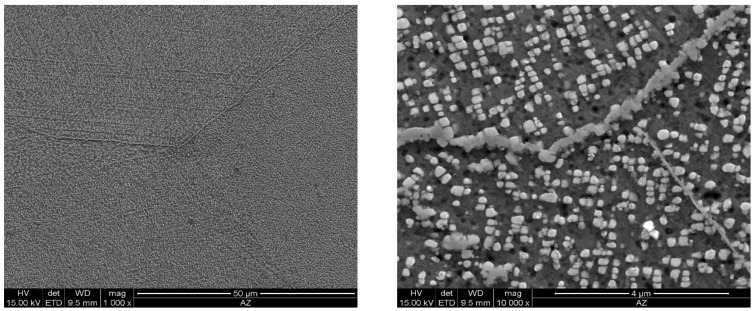
Scanning electron microscope analysis of the microstructure of Inconel 740H after ageing at 750 °C for 20,000 h.

**Figure 9:**
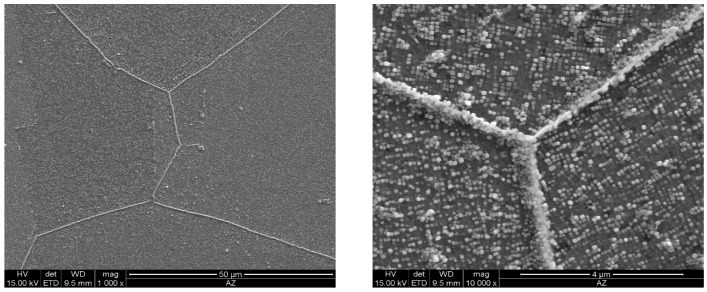
Scanning electron microscope analysis of the microstructure of Inconel 740H after ageing at 700 °C for 30,000 h.

**Figure 10:**
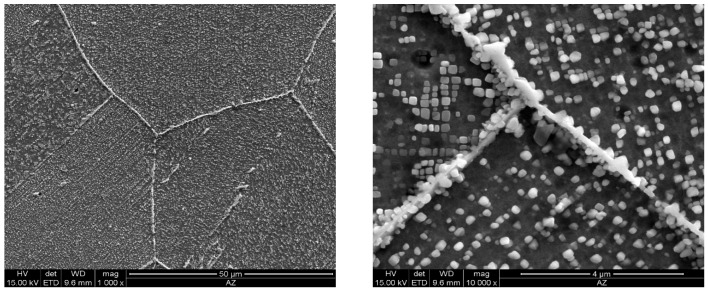
Scanning electron microscope analysis of the microstructure of Inconel 740H after ageing at 750 °C for 30,000 h.

**Figure 11:**
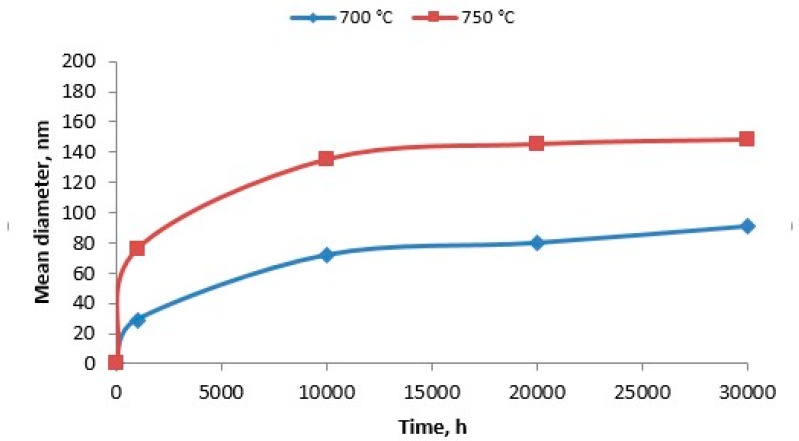
Change in the average diameter of γ’ phase depending on the time and temperature of ageing.

As the ageing time was increased to 10,000 h, an intense increase in the amount and size of Cr_23_C_6_ carbides and γ’ phase was observed. The Cr_23_C_6_ precipitated mainly at the austenite grain boundaries and at the twins, while the γ’ phase existed inside the austenite grains and in the immediate vicinity of carbides at the grain boundaries (Figure 3, Figure 4, Figure 5, Figure 6, Figure 7, Figure 8, Figure 9 and Figure 10). An increase in the precipitation process intensity was observed with an increase in temperature, as shown in the recorded microstructure images. During 1000 h of ageing at 700 °C (Figure 3), numerous very fine γ’ phase precipitates were visible. The assessment of its morphology required ca. 10,000× magnification. The increase in ageing temperature to 750 °C contributed to a significant increase in the volume of the γ’ phase (Figure 4). The average diameter of this phase was 100% greater than after ageing for the same time, but at a lower temperature (i.e., 700 °C; Figure 3). The ageing at 700 °C resulted in the greatest increase in the average diameter of the γ’ phase, until 10,000 h (Figure 5). Prolonging the time produced a further increase; however, at a much lower rate (Figure 7 and Figure 9). A similar trend was observed at 750 °C (Figure 6, Figure 8, and Figure 10), which was confirmed by the quantitative analysis of the precipitates. The quantitative analysis of the γ’ phase precipitates (Figure 11) showed that the average diameter of this phase, after ageing at 700 and 750 °C for 1000 h, was 29 and 76 nm, respectively. Whereas, after ageing at 700 and 750 °C for 10,000 h, it was 72 and 135 nm, respectively. Up to this point the highest growth rate of the γ’ phase was observed. However, further ageing up to 30,000 h resulted in an insignificant increase in the γ’ phase, with a mean diameter of 91 and 148 nm at 700 and 750 °C, respectively. This resulted in a 20% and 9% increase, respectively, compared to the measured values after the ageing time of 10,000 h.

**Figure 12:**
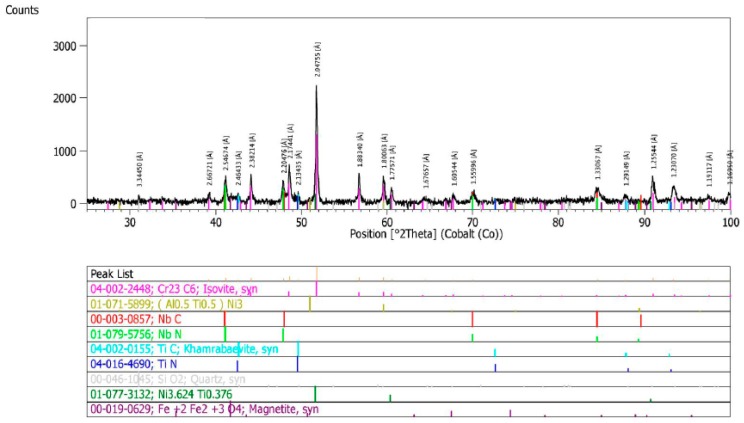
X-ray diffractogram of precipitate isolate in Inconel 740H after ageing at 700 °C for 1000 h.

**Figure 13:**
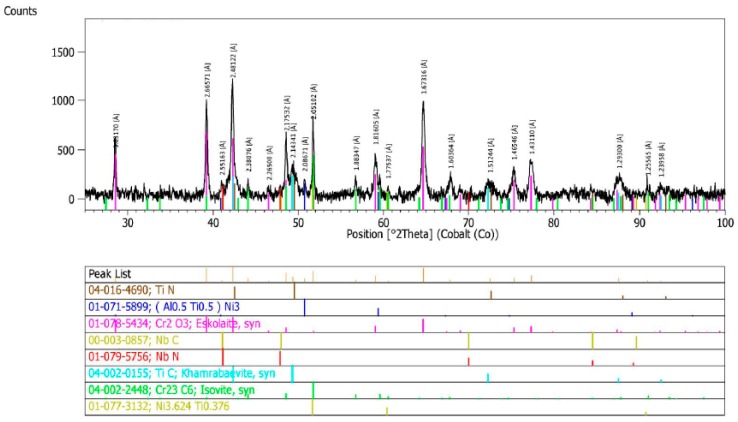
X-ray diffractogram of precipitate isolate in Inconel 740H after ageing at 750 °C for 1000 h.

X-ray phase analysis of the precipitates in the test alloy, after ageing at 700 °C for 1000 h, showed that the main phase was Cr_23_C_6_ carbide and, to a lesser extent, the γ’ phase (Figure 12). Raising the ageing temperature to 750 °C significantly intensified the precipitation process of the γ’ phase, which showed the highest percentage after a period of 1000 h (Figure 13) [19,20].

**Figure 14:**
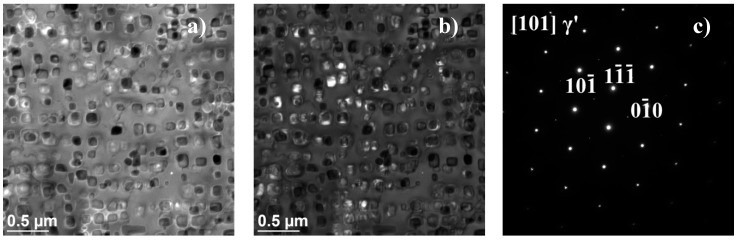
γ’ phase precipitation in Inconel 740H after ageing at 750 °C for 10,000 h, using TEM: (**a**) Bright field; (**b**) dark field; and (**c**) resolved diffractogram.

**Figure 15:**
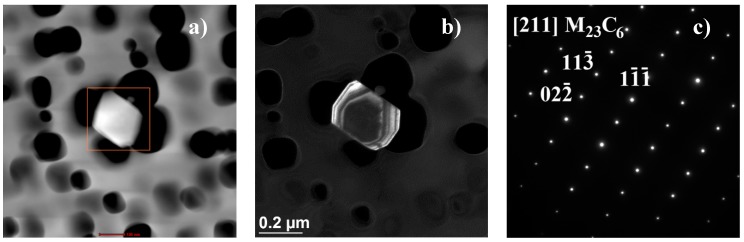
Cr_23_C_6_ carbide precipitation observed inside the austenite grain in Inconel 740H after ageing at 750 °C for 1000 h, using TEM: (**a**) Bright field, (**b**) dark field, and (**c**) resolved diffractogram.

**Figure 16:**
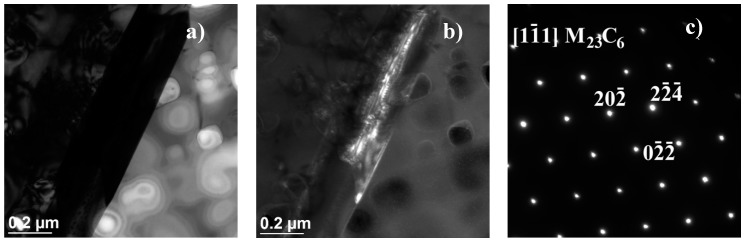
Cr_23_C_6_ carbide precipitation observed at the austenite grain boundary in Inconel 740H after ageing at 750 °C for 1000 h, using TEM: (**a**) Bright field, (**b**) dark field, and (**c**) resolved diffractogram.

The results obtained above were further elaborated by studies on the microstructure, including the identification of precipitates, and their morphology, occurring in the test alloy, which were conducted by transmission electron microscopy [20]. In Inconel 740H after ageing at 750 °C for 10,000 h, the γ’ phase (Figure 14) were identified and Cr_23_C_6_ precipitates (Figure 15 and Figure 16). The chemical composition of the identified precipitates is summarized in Table 2.

### 3.2. Mechanical Properties

**Figure 17:**
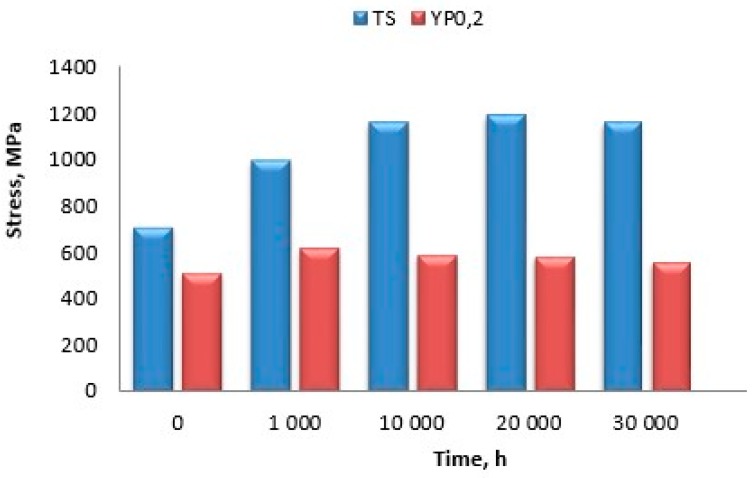
Strength properties of Inconel 740H after long-term ageing at 750 °C.

The long-term impact of elevated temperatures resulted in a change in the strength properties due to precipitation of secondary phases (γ’ and Cr_23_C_6_). Figure 17 shows the effect of the ageing time at 750 °C on tensile strength (TS) and yield point (YP_0.2_) determined at room temperature. A continuous increase in tensile strength was observed until an ageing time of 20,000 h, when it amounted to 42% relative to the as-received state of the test alloy. Following that time, a decrease in tensile strength by ca. 5% was observed. A ca. 17% increase in the yield point, relative to the as-received state, was observed until the ageing time of 1000 h; prolongation caused a decrease reaching 557 MPa after 30,000 h, which was still higher than that in the as-received state by 8%.

**Figure 18:**
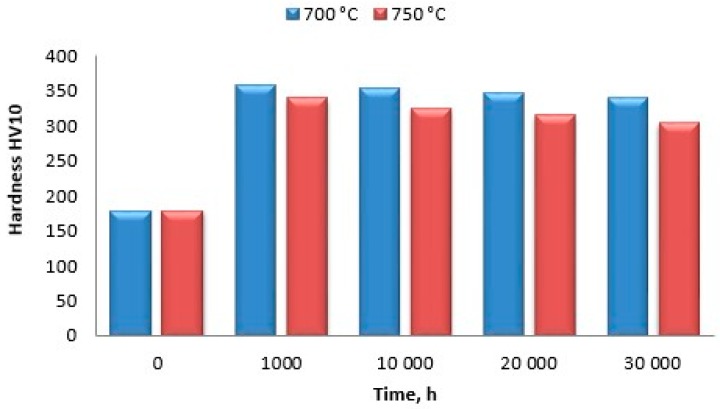
Change in hardness HV10 depending on time and temperature of Inconel 740H ageing.

Similar trends were observed for HV10 hardness measurements (Figure 18). At both 700 and 750 °C, the maximum increase in hardness was recorded after the ageing time of 1000 h, and then a slight yet continuous decrease was observed. After the ageing time of 30,000 h at 700 and 750 °C, the hardness HV10 increase was 44% and 40%, respectively.

## 4. Discussion

Long-term ageing of Inconel 740H affected the development of the precipitation processes, as well as the changes in morphology, coherent with the matrix of γ’ intermetallic phase and Cr_23_C_6_ carbides. This was evident from the recorded microstructure images (Figure 3, Figure 4, Figure 5, Figure 6, Figure 7, Figure 8, Figure 9 and Figure 10).

The main phase that hardened the Inconel 740H alloy was γ’ [Ni_3_(Al, Ti)], which was characterized by the same crystalline structure as the matrix but differed in the lattice parameter. The γ’ phase showed the highest lattice misfit with the matrix, whilst maintaining coherence, which was the major obstacle to dislocation movement during service under creep conditions. The unquestionable advantage of the γ’ phase was the enhancement of its strength with increased temperature without a reduction in ductility, which was a characteristic of other phases, such as carbides or carbo-nitrides. The degree of the γ/γ’ lattice misfit varied depending on the chemical composition of the γ’ phase as well as the chemical composition of the matrix, which also affected the morphology of the γ’ phase. The γ’ phase that contained more Ti was characterized by lower stability, compared to the γ’ phase that was rich in Al. During long-term service at elevated temperature, the Ti-rich γ’ phase may be transformed into η (Ni_3_Ti) phase, with a compact, hexagonal crystal structure and lamellar or needle-like morphology. Similar to the γ’ phase, the η phase does not exhibit coherence with the γ matrix and, at the same time, it is very brittle, adversely affecting the ductility [15].

The second phase, in terms of content phase, in Inconel 740H, occurring when it was exposed to elevated temperature, was M_23_C_6_ carbide. In nickel superalloys, these carbides are characterized by a significant Cr content with the possible existence of other elements (i.e., W, Mo, Ni, Co, and Fe). They either precipitated from the matrix or were a result of the transformation of the primary M_23_C_6_ carbide. Furthermore, the morphology of the M_23_C_6_ carbide had a significant impact on the plastic properties of nickel superalloys. Large M_23_C_6_ precipitates, in the form of continuous chains at the grain boundaries, had an adverse impact on the performance of the alloy, reducing its impact strength. Whereas fine M_23_C_6_ precipitates, in the form of single particles, contributed to an increase in creep strength, fatigue, and also enhanced crack resistance.

## 5. Conclusions

Due to its high creep strength at elevated temperatures and its very good high-temperature corrosion and steam oxidation resistance, the Inconel 740H alloy is recommended for long-term use under creep conditions in the temperature range 700–750 °C.

In the as-received condition (after solution annealing heat treatment), the test alloy was characterized by an austenitic microstructure, with visible annealing twins and an average austenite grain diameter of approximately 100 µm. X-ray phase analysis of the precipitates showed the presence of mainly primary niobium and titanium carbides and nitrides [Ni_3_(Al, Ti)] as well as trace amounts of Cr_23_C_6_ carbides and γ’ phase.

Investigations on ageing, at 700 and 750 °C for 1000, 10,000, 20,000, and 30,000 h, allowed for a complete view of the process of changes in the microstructure of Inconel 740H. This consisted mainly of its tendency to form γ’ as the main phase, and the unfavorable morphology of the Cr_23_C_6_ precipitates, forming both discontinuous and continuous carbide arrangements at the grain boundaries and annealing twins. These arrangements previously occurred in the early stages of ageing, up to 1000 h at 700 °C, when the thermally activated processes resulted in chromium segregation in micro-areas adjacent to the grain boundaries, followed by the formation of continuous, grid-like carbide arrangements.

Analysis of the increase in γ’ phase precipitates clearly indicated that the process of its growth was the most intense during the first 1000 h, and its growth rate slightly decreased towards 10,000 h. Following this time, further growth of the γ’ phase was very slow.

The ageing process conducted at these two temperatures also provided an opportunity to analyze the growth of the precipitates in the temperature range 700–750 °C. This temperature rise contributed to more than a tenfold increase in the rate of γ’ growth. This allowed the service life of the components, made of Inconel 740H and operated under creep conditions, to be estimated based on the analysis of their microstructure.

Investigations into the strength properties of Inconel 740H after ageing at 750°C, including tensile strength and yield points at room temperature, revealed the occurrence of significant precipitation hardening in the initial ageing period, up to 1000 h. Following this time, a very slow yet continuous decrease in tensile strength and yield point was observed, but with values still greater than those of the as-received material. Similar changes as a result of long-term service were confirmed by hardness measurements. The observed trend may indicate that the temperature of 750 °C is the maximum temperature for the long-term service of components made of Inconel 740H.

As part of the investigations described above, ageing tests up to 50,000 and 100,000 h were also performed. Results will be published after the intended ageing times are reached.

## Figures and Tables

**Table 1:** Chemical composition of the investigated alloy (in wt. %).

	C	Mn	Cr	Ni	Ti	Mo	Co	Nb	Al
**Test material**	0.03	0.10	24.37	REST	0.87	1.56	20.67	0.65	1.44
**To UNS N07740**	0.005 0.08	≤1.0	23.50 25.50	REST	0.50 2.50	≤2.0	15.00 22.00	0.50 2.50	0.20 2.00

**Table 2:** Analysis of the chemical composition of the identified precipitates in Inconel 740H after 10,000 h ageing at 750 °C.

Analyzed Precipitate	Chemical Composition, Weight %							
	Al	Ti	Cr	Co	Ni	Mo	Nb	C
**γ’ phase**	4.14	6.18	2.00	4.92	73.66	-	9.30	-
**Cr_23_C_6_ (Figure 15)**	-	-	85.34	2.19	4.67	1.19	-	6.58
**Cr_23_C_6_ (Figure 15)**	-	-	85.37	1.91	3.94	1.6	-	8.16
